# An Incentive Solution to the Peer Review Problem

**DOI:** 10.1371/journal.pbio.0050107

**Published:** 2007-04-17

**Authors:** Marc Hauser, Ernst Fehr

Every researcher knows the experience: you send a manuscript in for review and it disappears into the ether as you wait, a painfully long time, for the review. As scientists, we want immediate gratification: either accept or reject. This enables us to move on with our work, building on prior findings. Alas, the review process doesn't work this way, and from our perspective, seems to have deteriorated further over the years, even with the improvements that the Internet has brought forward. Some people review quickly, while others do not. And yet, there are no rewards for the swift or punishments for the slackers. We would like to propose a solution based on the logic of incentives to stimulate discussion, while also appreciating the complexities of incentive provisions. As such, we see this letter as an opening card in a game that we hope will help us attain a better equilibrium, one from which an improved system of incentives for refereeing emerges.

All journal editors maintain databases of reviewers and, thus, have details of their histories, including when an individual received a manuscript for review, the suggested deadline for review, and the date of the submitted review. Further, all journals have requirements for a timely review. Here's a proposed solution to the problem that some individuals review swiftly and others, extremely slowly. Whenever a reviewer sends in a review, the date is logged, as is common practice. Next to the date the editor enters a positive or negative value that indicates the relative timeliness of the review: negative values for reviews arriving before the deadline, and positive values for those arriving afterwards. Reviewers that turn in their reviews late are punished, whereas those that arrive on time are rewarded. To make the punishments count, and hopefully curtail future transgressions, we recommend the following policy for reviewers who turn in their reviews late: for every day since receipt of the manuscript for review plus the number of days past the deadline, the reviewer's next personal submission to the journal will be held in editorial limbo for twice as long before it is sent for review. To illustrate, consider a reviewer who is given three weeks to review and turns his review in two weeks after the deadline. The total review time for this reviewer is five weeks. The punishment is 10 weeks, meaning that his next submission sits in the editorial office for ten weeks before being sent out for review. Journals reward timely reviewers by sending their manuscripts out for review as soon as they come in, and if accepted, by pushing their papers high up into the publication queue.

Several problems immediately suggest themselves, and we address some of these here. First, in the case of multi-authored papers, only the primary corresponding author should trigger a punishment or reward assignment.

Second, though we realize that the timely reviewer is only gaining what should be the normal state of affairs, our intuition is that it is the cost to the negligent reviewer that is most important. Given the documented benefits of punishment in a variety of cooperation games, we imagine that this policy might well speed up the review process and curtail the number of slackers.

Third, if a given reviewer is the kind of person who rarely gets reviews in on time, he or she could exploit this system by simply refusing to review, or by sending in a less than helpful review, thereby avoiding the costs altogether. To close this loophole, we would add a further cost: for every manuscript that a reviewer refuses to review, we add on a one-week delay to reviewing their own next submission. Thus, if a reviewer rejects two consecutive manuscripts for review, his next submission sits in editorial limbo for two weeks. As for unhelpful reviews, these occur even with the present system, and it is an empirical question as to whether the rates would increase under the proposed review process. If they did, we would propose some minimal criteria for a useful review; anything less would be subject to the same penalty as proposed for opting out of review. For reviewers who opt out or turn in insufficient reviews, the only way to break the cycle of penalties is by providing a substantive review for the journal.

Fourth, journals may worry that by implementing this policy, they might lose manuscripts from some of the more interesting scientists, who may happen to be slow reviewers; we fully recognize the distinct possibility that there probably isn't a strong positive correlation between the quality of scientific research and the timeliness of reviews. But given the hierarchies among journals in every field, as well as the diversity of options, we don't expect this to be a significant problem for most journals.

For the proposed system to work, the journals must fully commit to this policing policy. Journal editors may sometimes be tempted to violate this policy, in order to clear the manuscript table, but this will not influence the status of a reviewer. In essence, editors must punish wrongdoers, full stop.

As humans, we are highly sensitive to rewards and punishments, perhaps not as exquisitely as rats in the proverbial Skinner box, but close enough. Clearly, the review process is broken. It is time to consider a fix. We have proposed a solution based on the logic of economic incentives and the evolutionary origins of human nature.

